# Supplementary material for: Targeting of NLRP3 inflammasome with gene editing for the amelioration of inflammatory diseases
Source: Nat Commun. 2018 Oct 5;9:4092. doi: 10.1038/s41467-018-06522-5 (PMC6173702; doi:10.1038/s41467-018-06522-5)
Supplement: Supplementary file 1 — Supplementary Information [file 41467_2018_6522_MOESM1_ESM.pdf]

Supplementary Information

Targeting of NLRP3 inflammasome with gene editing for the amelioration of inflammatory diseases

Xu et al.

Supplementary Table 1. The components for preparation of CLANs encapsulating Cy5-siRNA.

Components	CLAN number	Cationic Lipid-Assisted Polymeric Nanoparticles (CLAN)															
	Feeding weight	11	12	13	14	21	22	23	24	21	32	33	34	41	42	43	44
PEG_{5K}-*b*-$PLGA_{11K}$(mg)		25	21.9	20.3	18.8	25	21.9	20.3	18.8	25	21.9	20.3	18.8	25	21.9	20.3	18.8
$PLGA_{11K}$(mg)		0	2.1	3.2	4.3	0	2.1	3.2	4.3	0	2.1	3.2	4.3	0	2.1	3.2	4.3
Mass fraction of PEG_{5K}-*b*-$PLGA_{11K}$(%)		100	91.3	86.4	81.4	100	91.3	86.4	81.4	100	91.3	86.4	81.4	100	91.3	86.4	81.4
BHEM-Chol (mg)		1	1	1	1	1.5	1.5	1.5	1.5	2	2	2	2	3	3	3	3
Cy5-siRNA (μg)		200	200	200	200	200	200	200	200	200	200	200	200	200	200	200	200

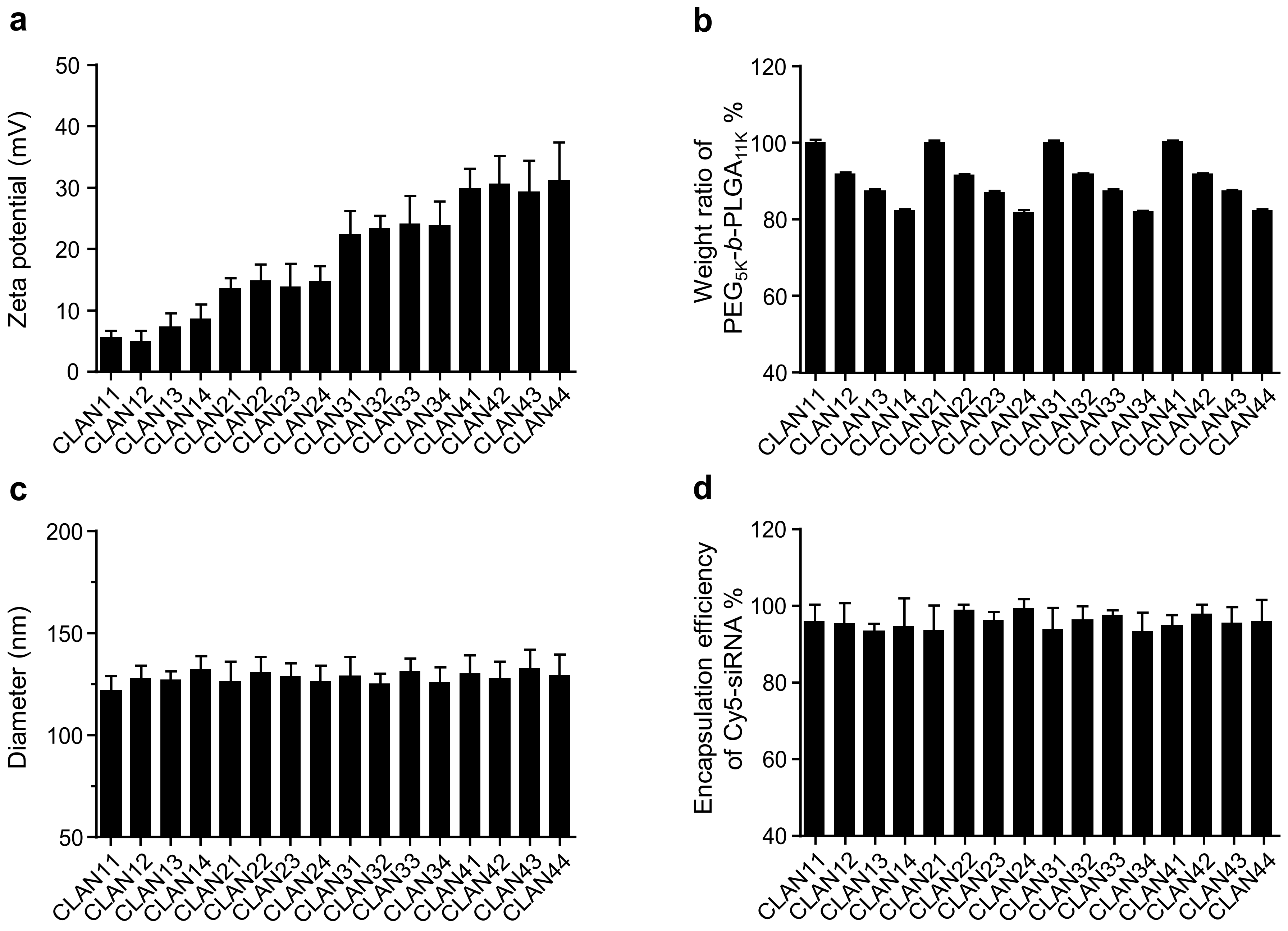

Supplementary Fig. 1 Characterization of CLANs encapsulating Cy5-siRNA. **a** Zeta potential of each CLAN. **b** Mass fraction of PEG_{5K}-*b*-$PLGA_{11K}$ in polymers of each CLAN. The percentage of PEG_{5K}-*b*-$PLGA_{11K}$ was characterized by ^{1}H NMR. **c** Diameter of each CLAN. **d** Encapsulation efficiency of Cy5-siRNA in each CLAN. The data are shown as the means ± SEM of n = 3 **a-d**.

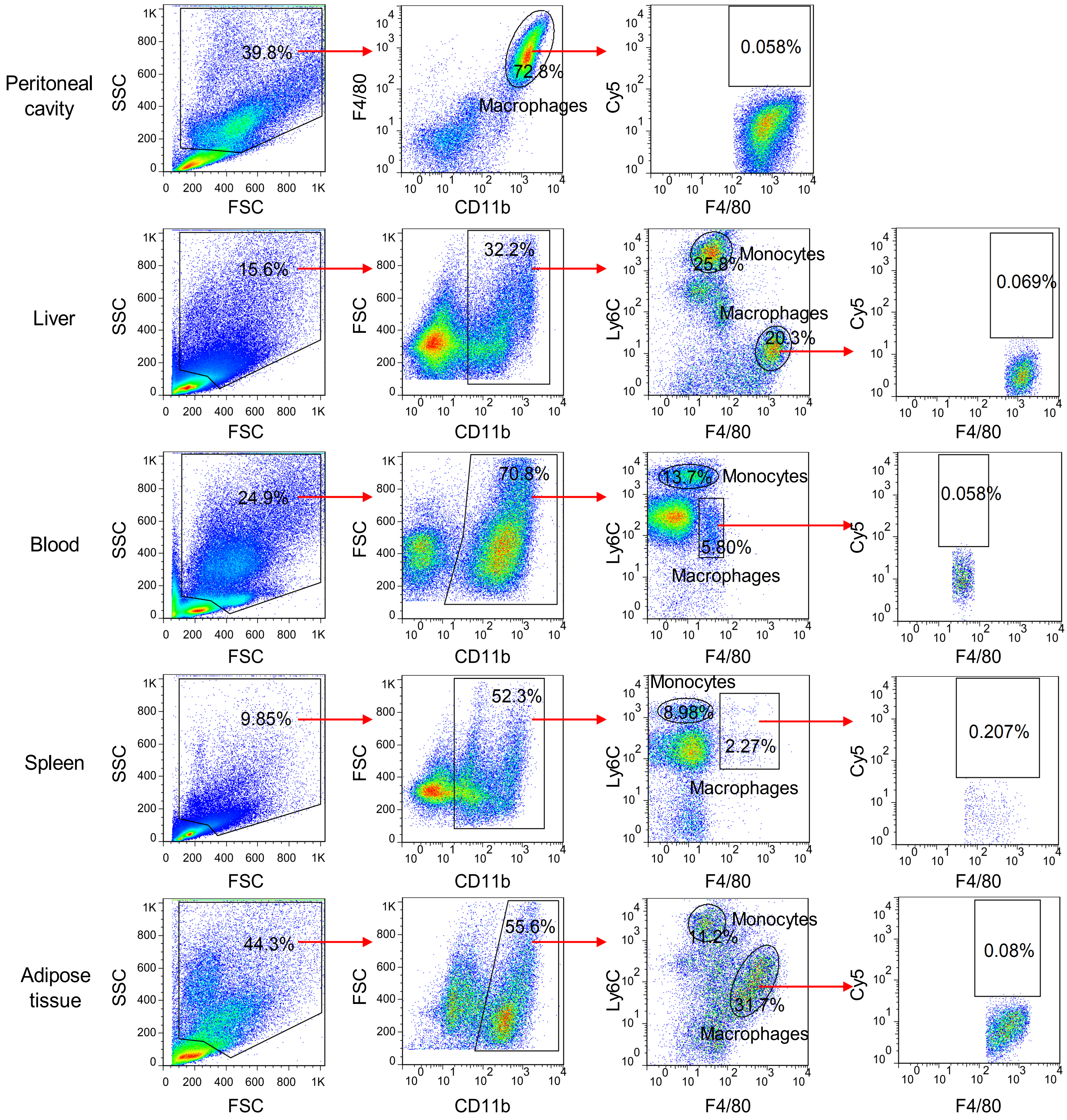

Supplementary Fig. 2 Gating Strategies of macrophages in the peritoneal cavity, liver, peripheral blood, spleen and adipose tissue. All macrophages ($CD11b^{+}F4/80^{+}$) from C57BL/6 mice were assessed by flow cytometry.

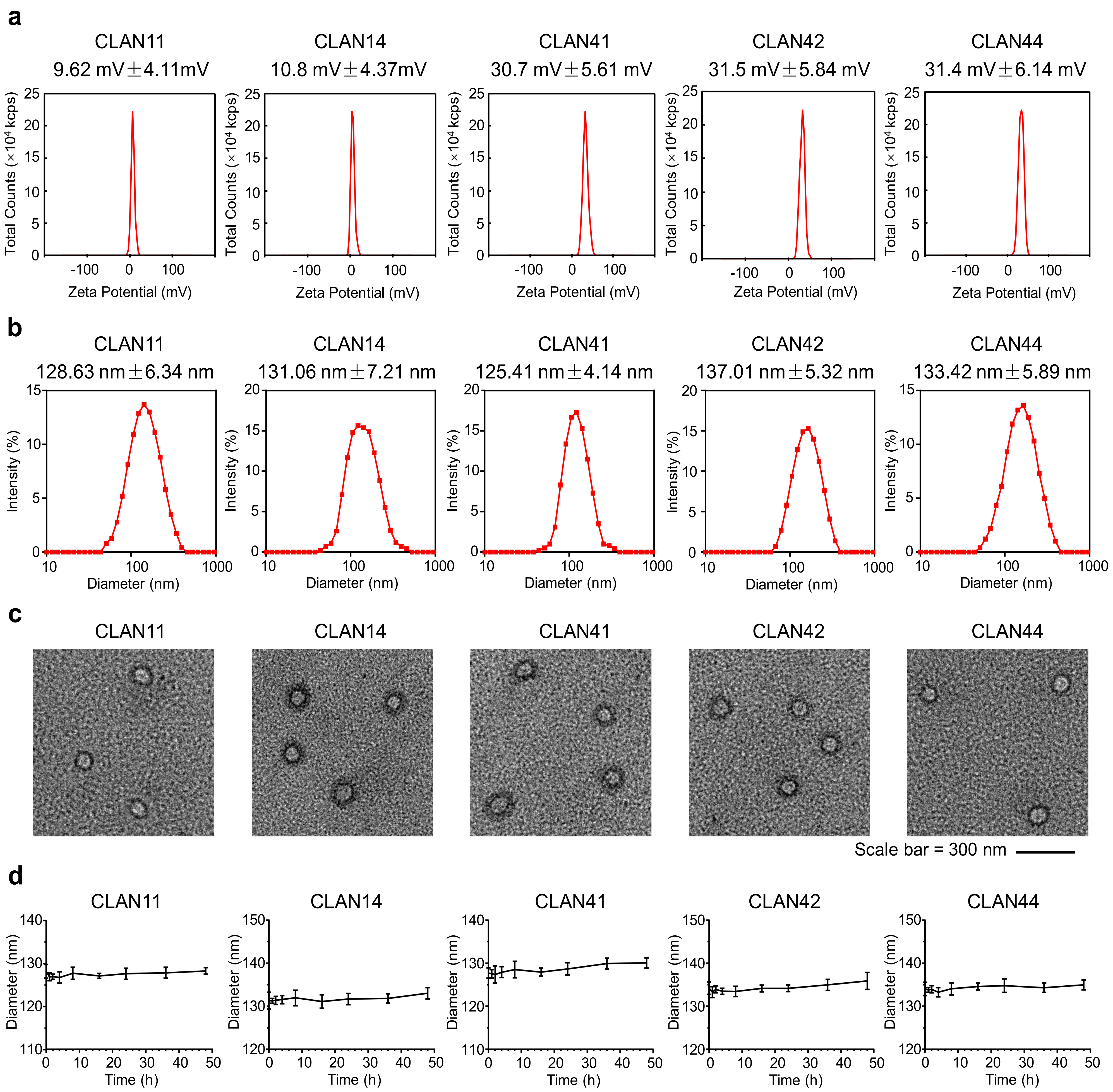

Supplementary Fig. 3 Characterization of CLANs encapsulating mCas9/gRNA. **a** Zeta potential of each CLAN$_{mCas9/gRNA}$. **b** Diameter of each CLAN$_{mCas9/gRNA}$. **c** Morphology of each CLAN$_{mCas9/gRNA}$ analyzed by transmission electron microscopy (TEM). **d** Colloidal stability test of each CLAN$_{mCas9/gRNA}$ following incubation with PBS containing 10% FBS at 37 °C. Data are shown as the means ± SEM of n = 3 **a-b, d** or are representative of two independent experiments **c**.

Supplementary Table 2. Encapsulation efficiency and loading efficiency of mCas9/gRNA in CLANs.

	CLAN11	CLAN14	CLAN41	CLAN42	CLAN44
Encapsulation efficiency %	93.9 ± 0.1	94.5± 0.5	95.1± 0.4	96.1 ± 0.3	95.4 ± 0.2
Loading efficiency %	52.0 ± 2.1	54.8 ± 2.7	55.1± 3.2	54.2 ± 2.6	52.3 ± 1.4

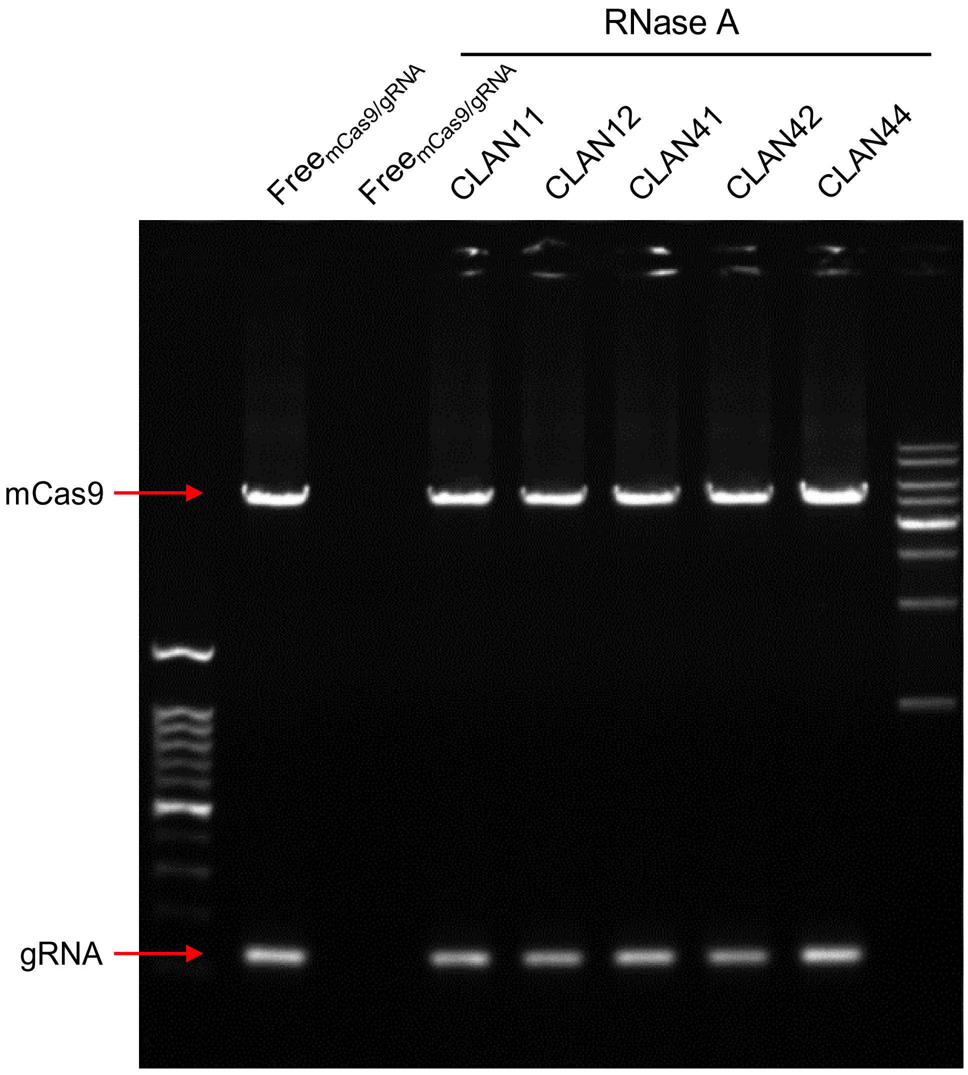

Supplementary Fig. 4 Integrity of mCas9/gRNA in CLANs. CLANs encapsulating mCas9/gRNA were treated with RNase A (20 µg mL^{-1}) for 4 h at 37 °C before RNA extraction for denaturing gel electrophoresis. Free mCas9/gRNA (Free$_{mCas9/gRNA}$) treated with or without RNase A were used as control. The data are representative of three independent experiments.

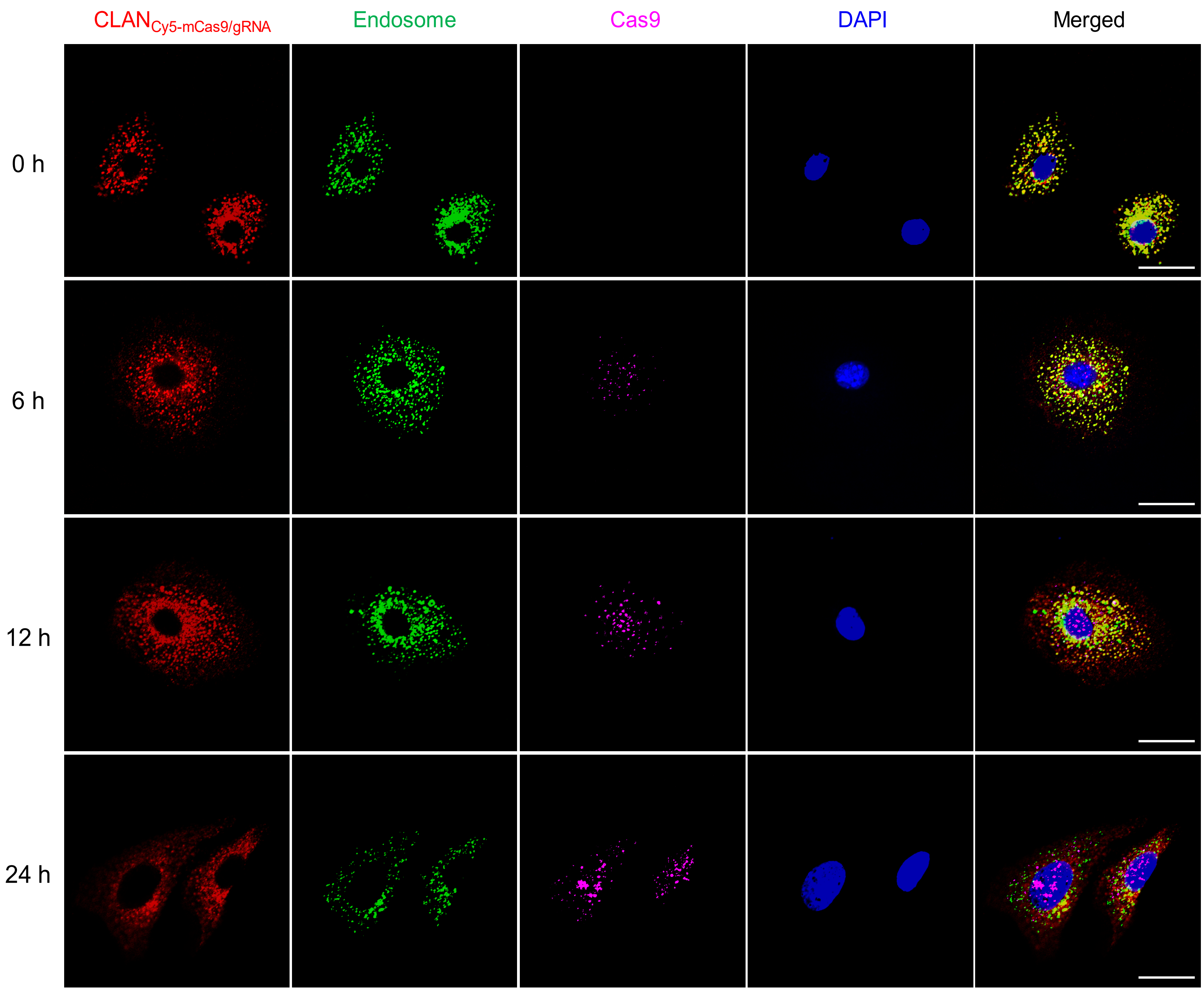

Supplementary Fig. 5 Analysis of the localization of $CLAN_{Cy5\text{-}mCas9/gRNA}$ and Cas9 protein at different time points after transfection. BMDMs were incubated with $CLAN_{Cy5\text{-}mCas9/gRNA}$ for 6 h at the concentration of 2.6 nM Cy5-Cas9 mRNA. Endosome was marked with Alexa Fluor 488-labeled EEA1 antibody and Cas9 protein was marked with Alexa Fluor 568 -labeled Cas9 antibody. Scale bar = 20 μm. The data are representative of two independent experiments.

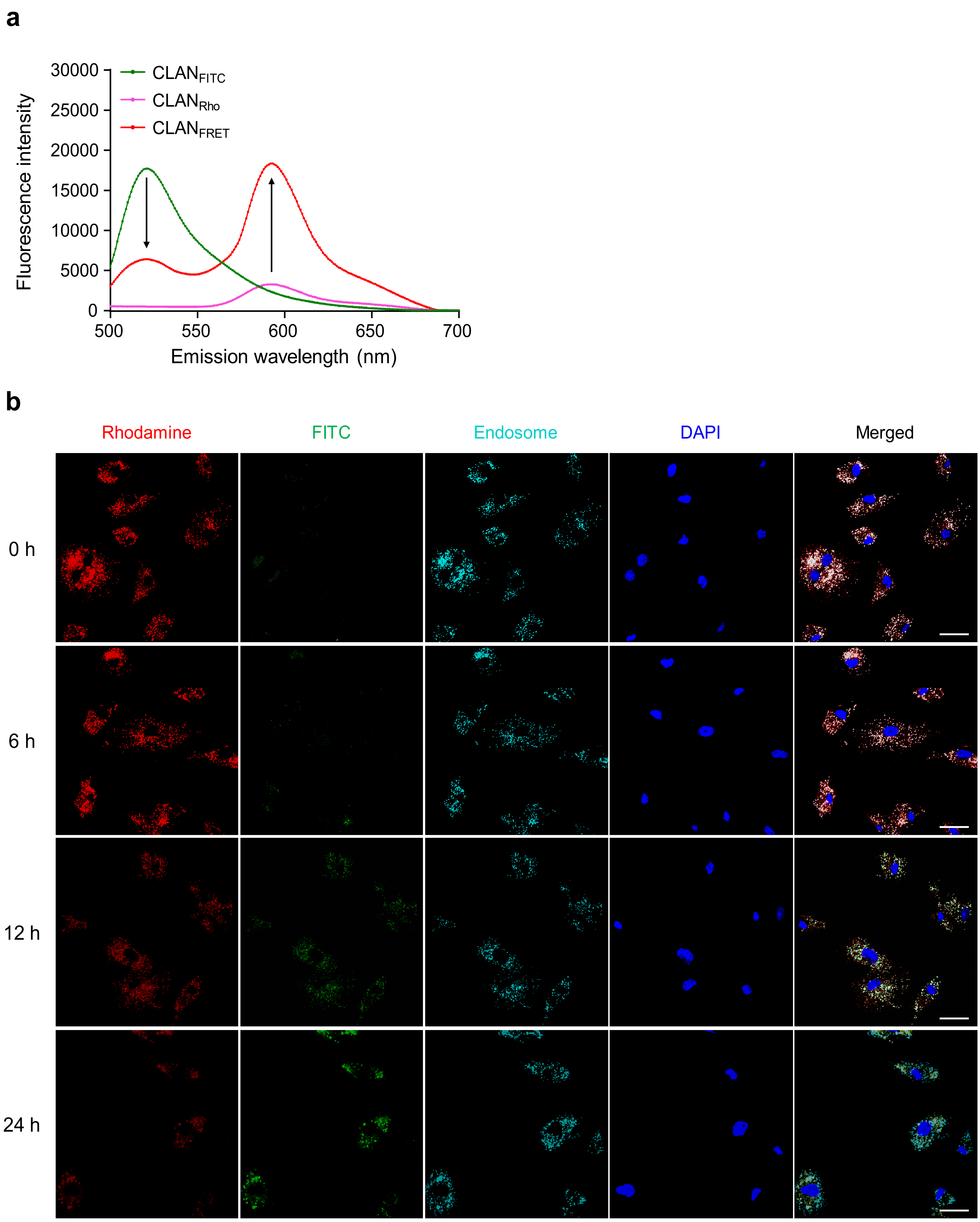

Supplementary Fig. 6 Analysis of the integrity of $CLAN_{FRET}$. **a** Detection of the fluorescence intensity of $CLAN_{FITC}$, $CLAN_{Rho}$ and $CLAN_{FRET}$ using RF-6000 fluorescence spectrophotometer with the excitation light of 488 nm. **b** Analysis of the integrity of $CLAN_{FRET}$ at different time points after transfection into BMDMs. BMDMs were incubated with $CLAN_{FRET}$ for 6 h at the concentration of 2.6 nM Cas9 mRNA. Endosome was marked with Alexa Fluor 647-labeled EEA1 antibody and nucleus was marked with DAPI. The fluorescence of FITC and Rhodamine were observed with confocal microscope using the excitation light of 488 nm. Scale bar = 20 μm. The data are representative of two independent experiments **a, b**.

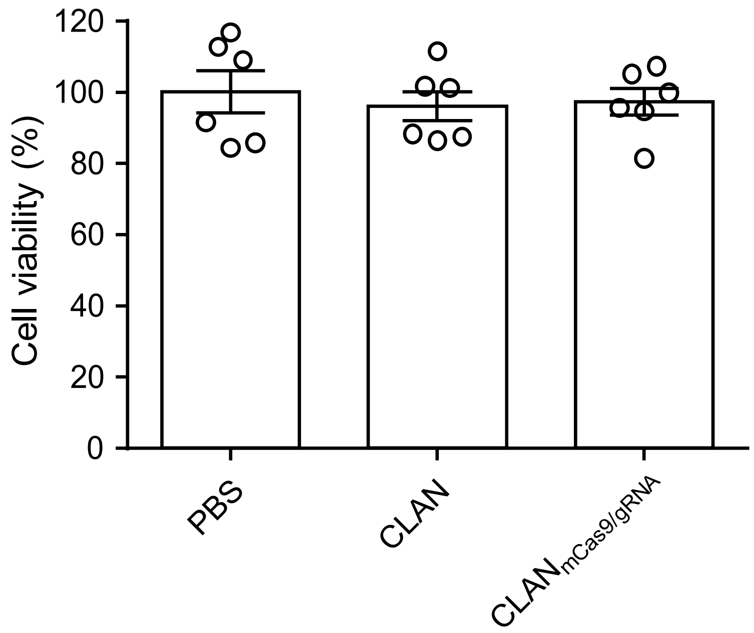

Supplementary Fig. 7 Detection of the cytotoxicity of CLAN$_{mCas9/gRNA}$ with MTT assay. BMDMs were incubated with CLAN$_{mCas9/gRNA}$ for 24 h at the concentration of 2.6 nM Cas9 mRNA. Then the cell viability of BMDMs were measured with MTT cytotoxicity assay. The data are shown as mean ± SEM of n = 6.

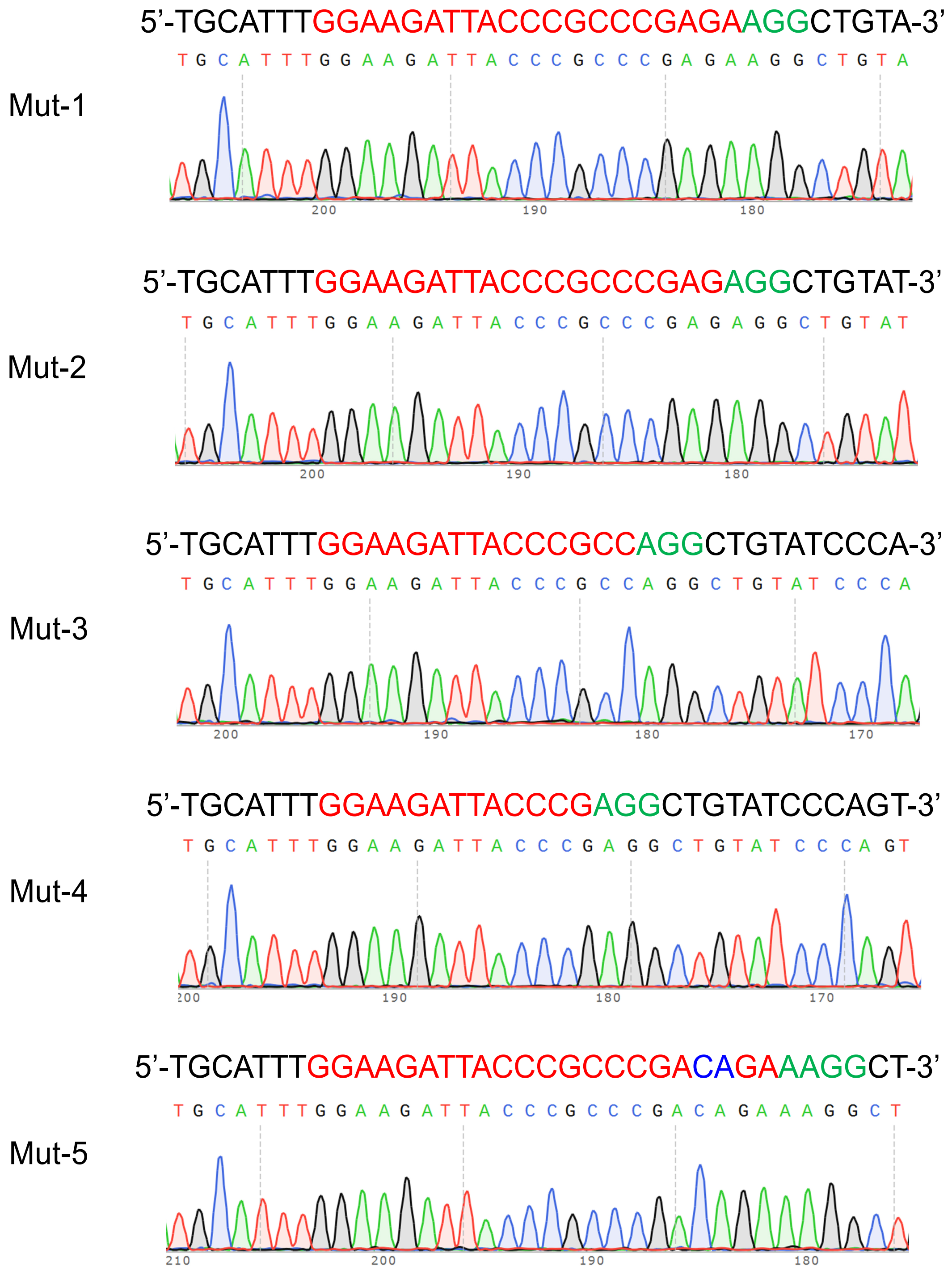

Supplementary Fig. 8 Representative sanger sequencing of indels (Mut) in the NLRP3 locus of BMDMs transfected with CLAN$_{mCas9/gNLRP3}$, related to **Fig.2c**. Target sequence was in red, PAM motif was in green and inserted bases were in blue.

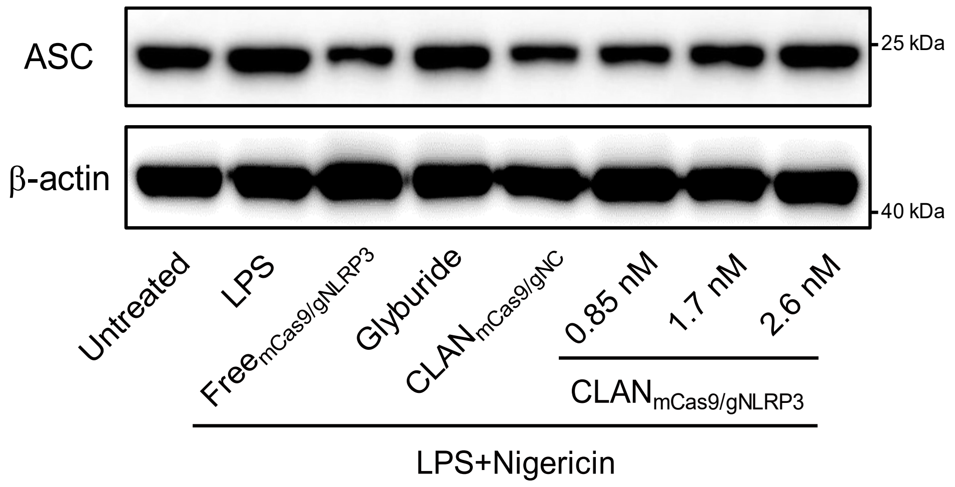

Supplementary Fig. 9 The ASC monomers expression in the cell lysate of BMDMs. BMDMs were transfected with CLAN$_{mCas9/gNLRP3}$ or treated with other formulations and stimulated with LPS and nigericin. The data are representative of two independent experiments.

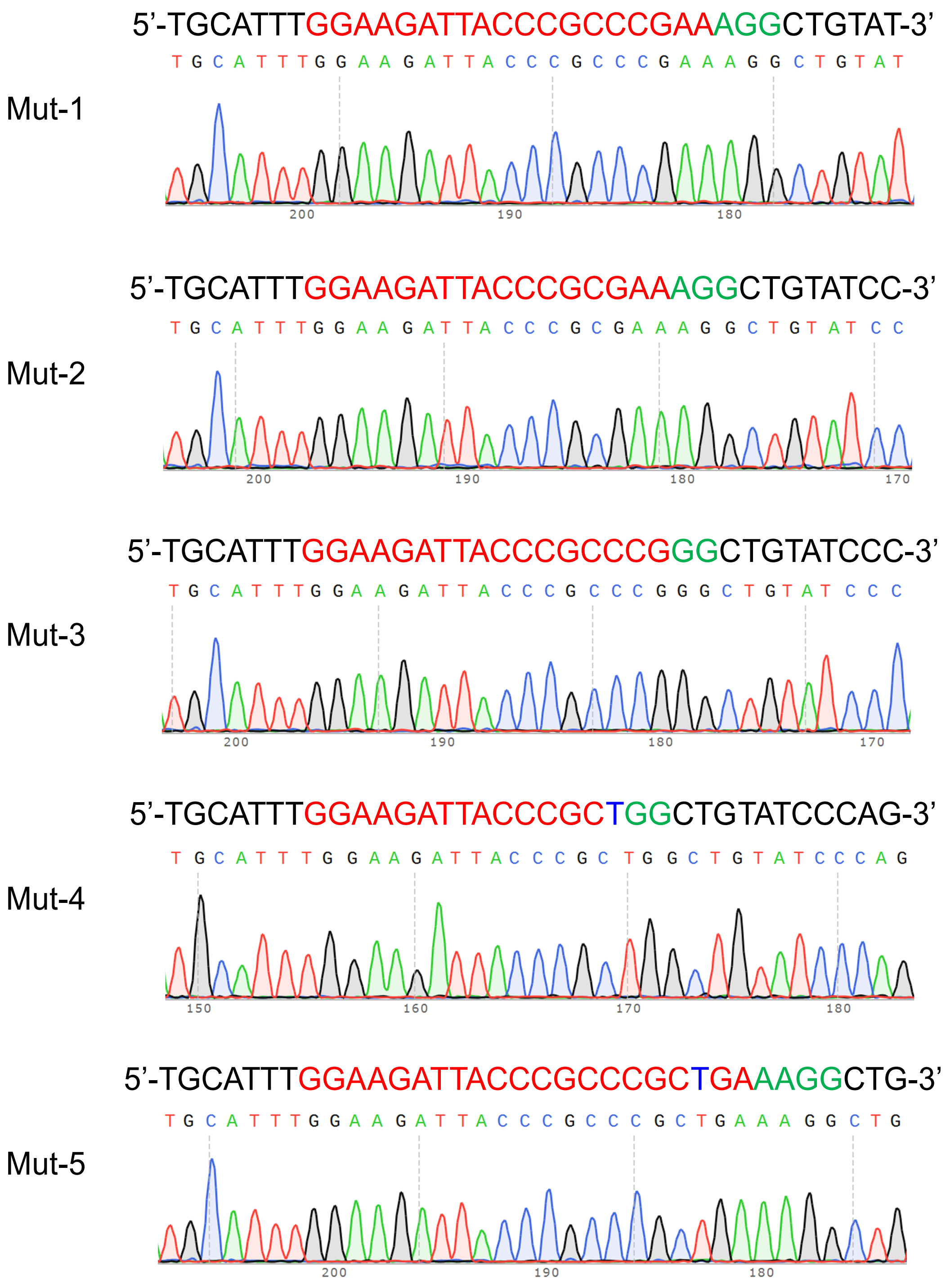

Supplementary Fig. 10 Representative sanger sequencing of indels (Mut) in the NLRP3 locus of peritoneal macrophages from mice injected with CLAN$_{mCas9/gNLRP3}$, related to **Fig. 4c**. Target sequence was in red, PAM motif was in green and inserted bases were in blue.

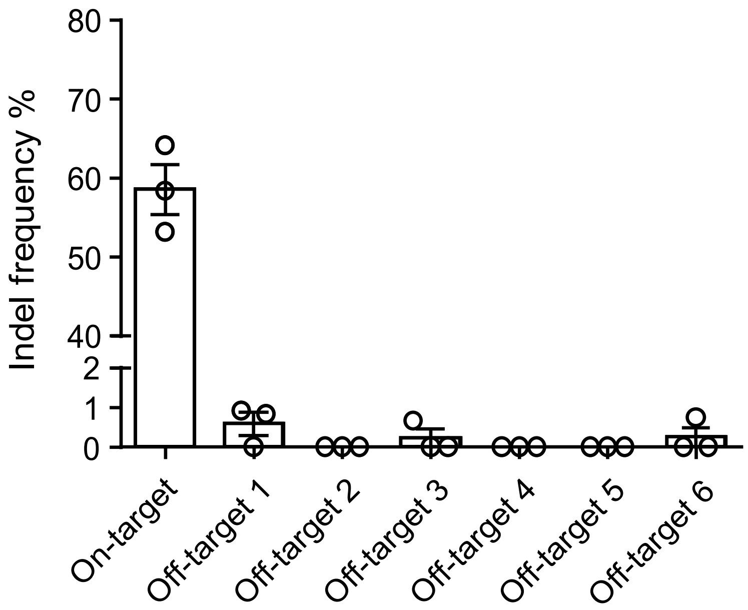

Supplementary Fig. 11 On-target (NLRP3) and off-targets indel frequency of peritoneal macrophages from mice injected with $CLAN_{mCas9/gNLRP3}$ (at the dose of 2 mg kg^{-1} total RNA). Genomic DNA of peritoneal macrophages were isolated 72 h after $CLAN_{mCas9/gNLRP3}$ injection and subjected to T7E1 assay for indel frequency detection. The data are shown as the means ± SEM of n=5.

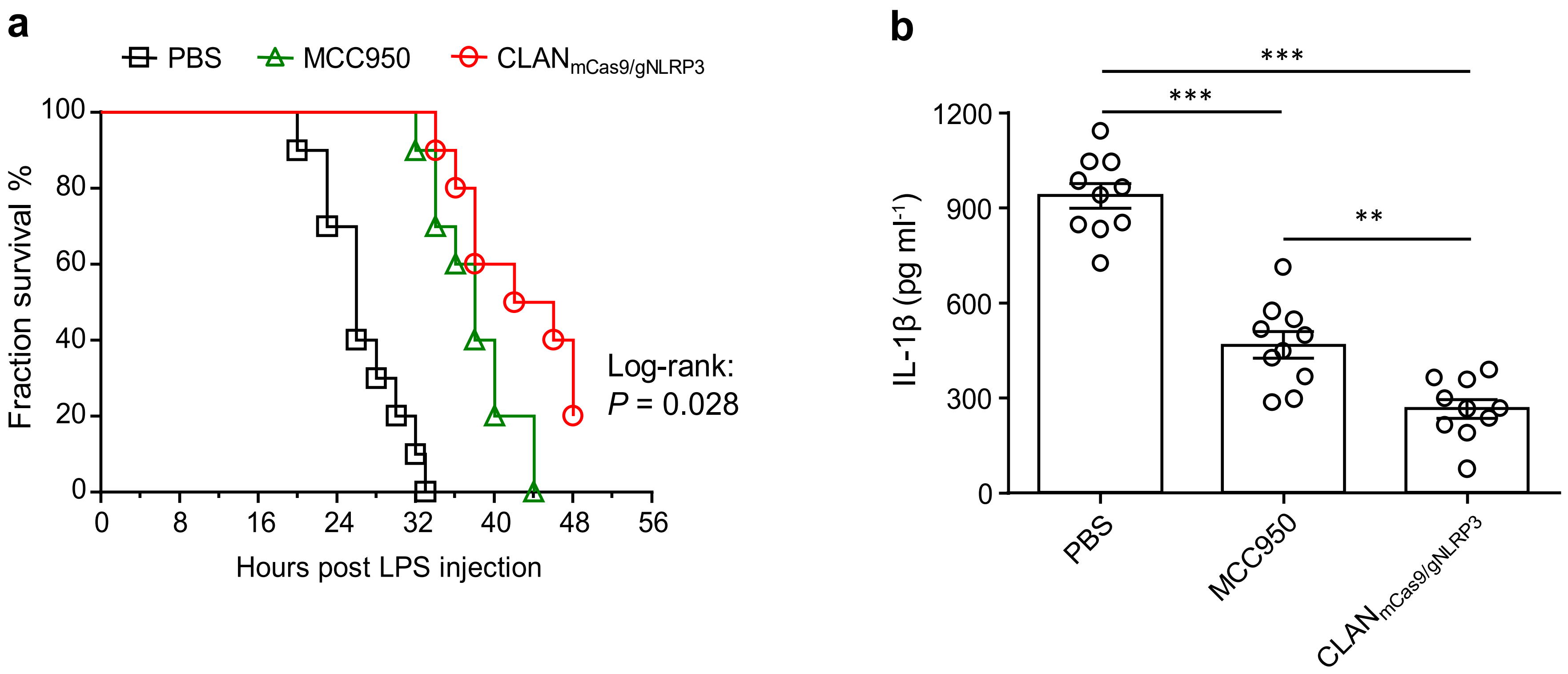

Supplementary Fig.12 Comparison of the efficacy of $CLAN_{mCas9/gNLRP3}$ and MCC950 for LPS-induced septic shock intervention. **a** The survival curve of LPS-induced septic shock mice. n = 10 per group. **b** Detection of the IL-1β concentration in the serum. C57BL/6 mice (6-8 weeks) were first injected daily with $CLAN_{mCas9/gNLRP3}$ (intravenous injection, 4 mg kg^{-1} total RNA) or MCC950 (intraperitoneal injection, 50 mg kg^{-1}) for three injections. Two days after the last injection, all mice were intraperitoneally injected with LPS (20 mg kg^{-1}) to induce septic shock. Serum was collected at 4 h after LPS induction. The data are shown as the means ± SEM of n = 10. Log-rank test **a** Two-way ANOVA **b** $^{**}P < 0.01$, $^{***}P < 0.001$.

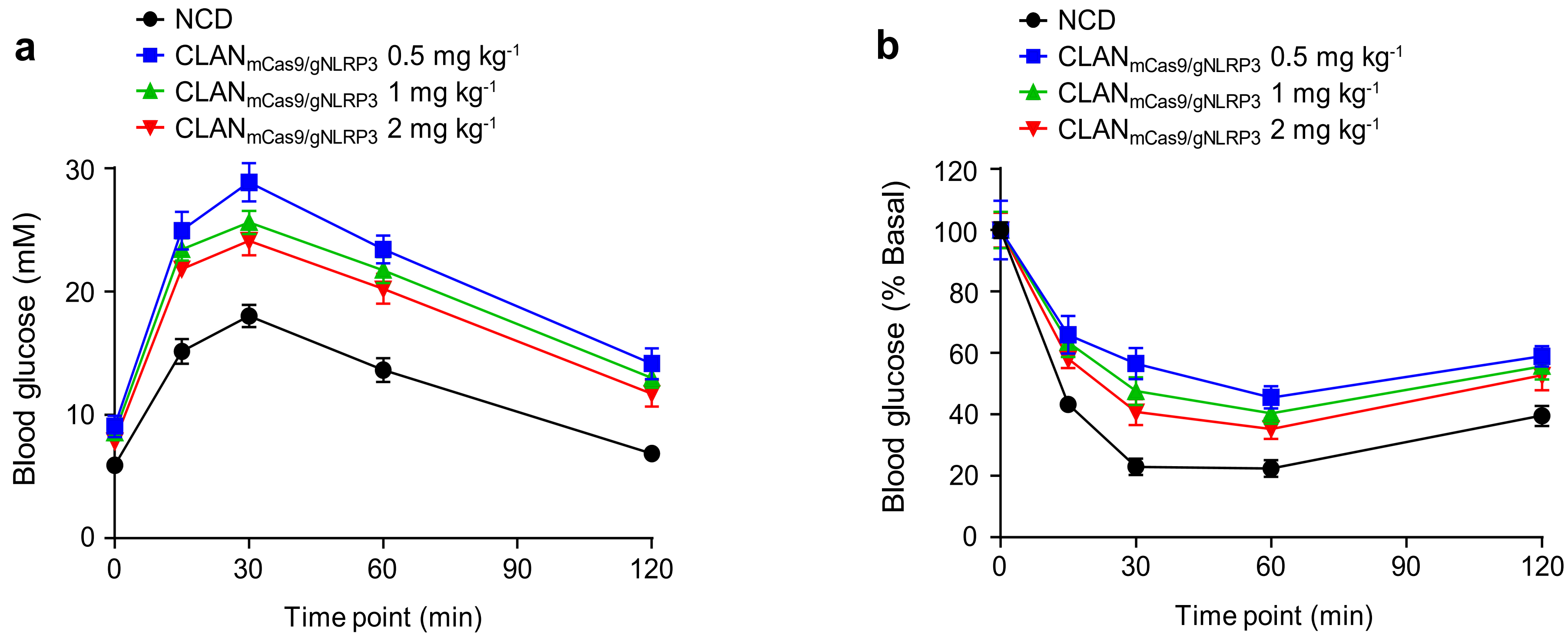

Supplementary Fig. 13 Glucose tolerance test (GTT) **a** and insulin tolerance test (ITT) **b** of HFD-induced T2D mice treated with CLAN$_{mCas9/gNLRP3}$ at the dose of 0.5 , 1 or 2 mg kg^{-1} total RNA, related to **Fig. 7c** and **Fig. 7d**. NCD, normal chow diet. The data are shown as the means ± SEM of n = 6-8 **a-b**.

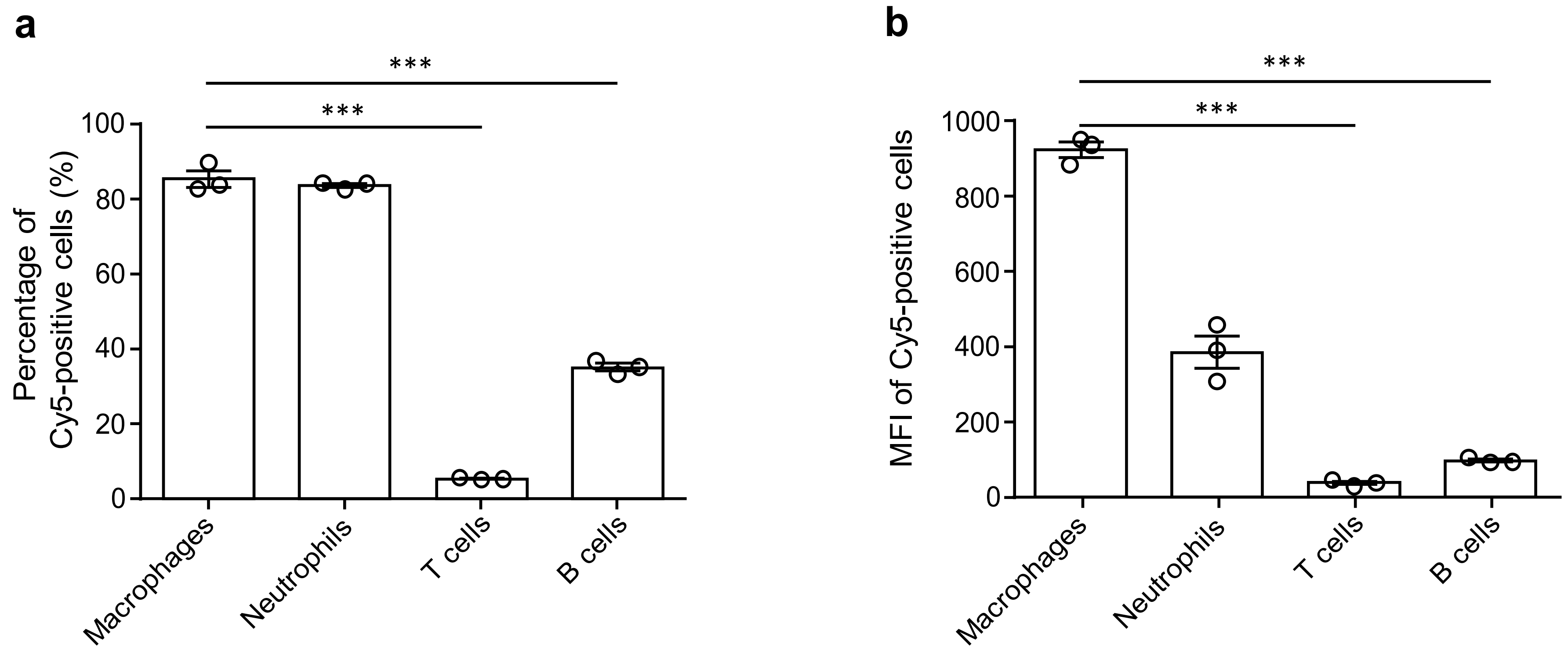

Supplementary Fig. 14 Detection of the cellular uptake of CLAN42 by different immunocytes of the blood. **a** Percentage of Cy5-positive cells of the blood. **b** MFI of Cy5-positive cells of the blood. CLAN42 encapsulating Cy5-siRNA (CLAN$_{Cy5\text{-}siRNA}$) was intravenously injected into C57BL/6 mice at the dose of 1 mg kg^{-1} Cy5-siRNA. Twenty-four hours later, the immunocytes of blood were isolated and analyzed by flow cytometry. The data are shown as mean ± SEM of n = 3. Two-way ANOVA **a, b** ***$P < 0.001$.

Supplementary Table 3. The components for preparation of CLANs encapsulating Cy5-siRNA.

Oligonucleotides ID	Sequence
NLRP3 gRNA oligo Forward	5'-CACCGAAGATTACCCGCCCGAGAA-3'
NLRP3 gRNA oligo Reverse	5'-AAACTTCTCGGGCGGGTAATCTTC-3'
GFP gRNA oligo Forward	5'-CACCGAGCTGGACGGCGACGTAAA-3'
GFP gRNA oligo Reverse	5'-AAACTTTACGTCGCCGTCCAGCTC-3'
Negative control gRNA oligo Forward	5'-CACCGCACTACCAGAGCTAACTCA-3'
Negative control gRNA oligo Reverse	5'-AAACTGAGTTAGCTCTGGTAGTGC-3'

Supplementary Table 4. Oligonucleotides used for making template for in vitro transcription (IVT).

Oligonucleotides ID	Sequence
Cas9 /Cas9-EGFP mRNA IVT F primer	5'-TAATACGACTCACTATAGGGAGAATGGACTATAAGGACCACGAC-3'
Cas9 mRNA IVT R primer	5'-GCGAGCTCTAGGAATTCTTAC-3'
Cas9-EGFP mRNA IVT R primer	5'-AGGCTGATCAGCGAGCTCTAG-3'
NLRP3 gRNA IVT F primer	5'-TTAATACGACTCACTATAGGGAAGATTACCCGCCCGAGAA-3'
GFP gRNA IVT F primer	5'-TTAATACGACTCACTATAGGGAGCTGGACGGCGACGTAAA-3'
Negative control gRNA IVT F primer	5'-TTAATACGACTCACTATAGGGCACTACCAGAGCTAACTCA-3'
gRNA IVT R primer	5'-AAAAGCACCGACTCGGTGCC-3'

Supplementary Table 5. Oligonucleotides used for T7E1 assay and indels sanger sequencing assay.

Oligonucleotides ID	Sequence
NLRP3 T7E1 assay F primer	5'-CCTGCACTGCCAGTGTGGACCTAAG-3'
NLRP3 T7E1 assay R primer	5'-GTTGGGAGCTTCAGTTGTGCAAGAT-3'
NLRP3 indels sequencing F primer	5'-CCCTCGAAAGGGCTGCTGCTGAAG-3'
NLRP3 indels sequencing R primer	5'-TCTCGCCTGTTGATCGCAGCAAAG-3'

Supplementary Table 6. On-target and potential off-targets of NLRP3 gRNA. Mismatches in red and PAM motif in green.

Targets	Sequence	Score	Mismatches	UCSC gene	Locus
On-target	5’-GAAGATTACCCGCCCGAGAAAGG-3'	100		NM_145827	chr11: +59356695
Off-target 1	5’-TGAGATTGCCCGCCTGAGAAAGG-3'	0.4	4MMs [1:2:8:15]	NM_020567	chr13: -24845271
Off-target 2	5’-GAATCTTACCTGTCCGAGAAAAG-3'	0.3	4MMs [4:5:11:13]	NM_001198955	chr1: +172236397
Off-target 3	5’-GAAGCTCACCTGCCCGAGGAAGG-3'	0.2	4MMs [5:7:11:19]	NM_001145935	chr4: -118843584
Off-target 4	5’-AAAGATCACCCCCCCCAGAAAAG-3'	0.1	4MMs [1:7:12:16]	NM_001198877	chr2: -71085310
Off-target 5	5’-GAAGATGACCTGCCAGAGACAGG-3'	0.1	4MMs [7:11:15:20]	NM_175308	chr4: -115503809
Off-target 6	5’-GACGATTACCCGCGGGAGGAGGG-3'	0.0	4MMs [3:14:15:19]	NM_023485	chr4: -128964813

Supplementary Table 7. Oligonucleotides used for amplifying off-targets of NLRP3 gRNA with PCR.

Oligonucleotides ID	Sequence
Off-target 1 F primer	5'-CTAAGGGAACCGAGTGAGGGAATGA-3'
Off-target 1 R primer	5'-GTCAGATAAAGTCACAAAGCCTATT-3'
Off-target 2 F primer	5'-GTTTGCCATACCCCTGTGTAGCAGT-3'
Off-target 2 R primer	5'-CTTGTGAAAACAGCCTCAGAAGGCA-3'
Off-target 3 F primer	5'-GTTATTCCTGGGGGACGGTATACAC-3'
Off-target 3 R primer	5'-CATTGGTAGTGCTTCTCTTCCAGGT-3'
Off-target 4 F primer	5'-GAATATGTGGCATTTCTTGAACCAT-3'
Off-target 4 R primer	5'-CTTACCTGGGATGACCAATCCAAAG-3'
Off-target 5 F primer	5'-CTATGGGGCTTCCAGAGGATACCTT-3'
Off-target 5 R primer	5'-CTTATATAGCTCAAAGCGCTGTGTG-3'
Off-target 6 F primer	5'-TCATGACAGCTGCAGGCCACCAA-3'
Off-target 6 R primer	5'-GGCTCGCCCGGAGTGTTATTAA-3'

Supplementary Fig. 15 Scans of the films used to generate blots data for Fig. 2a, 2b and 2d.

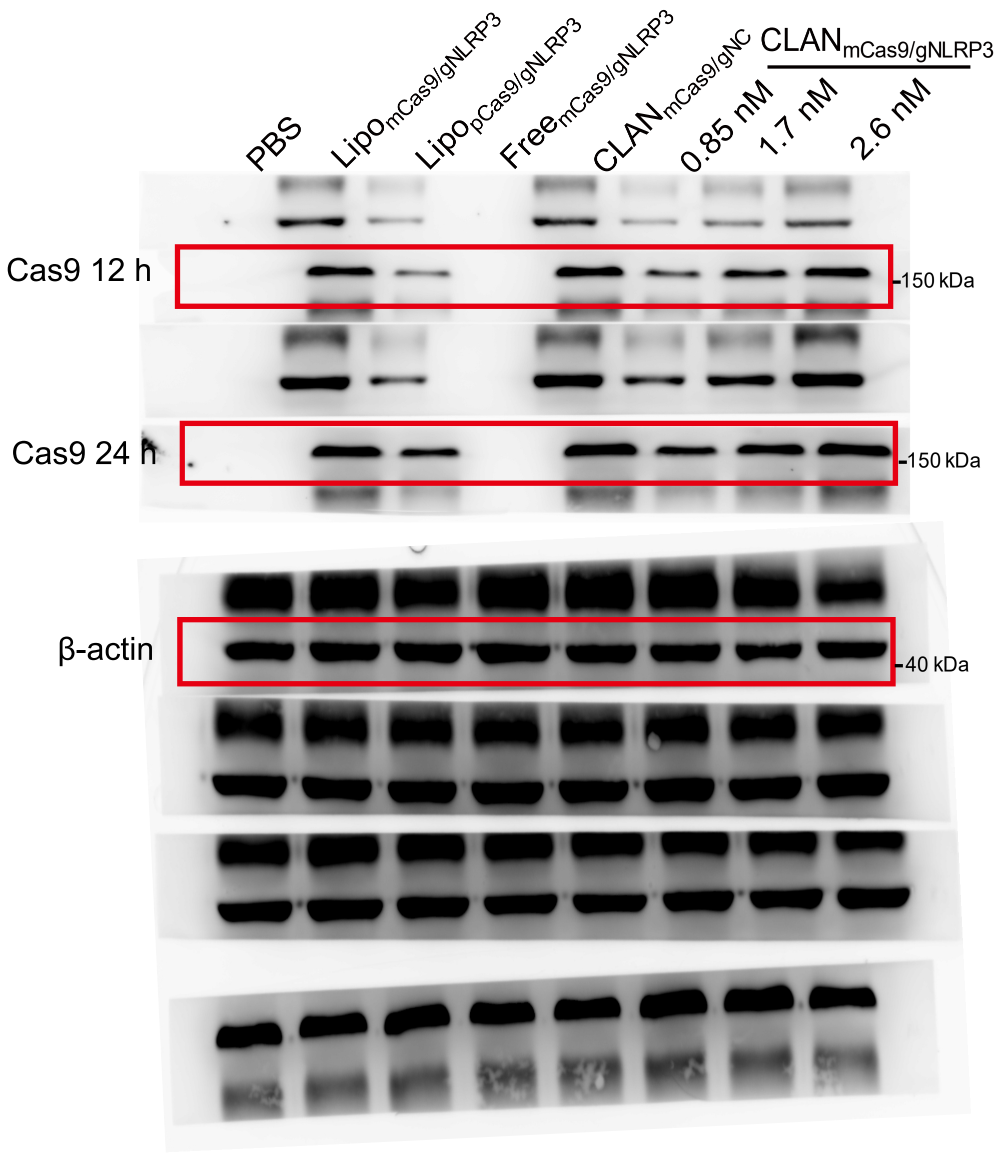

Fig. 2a

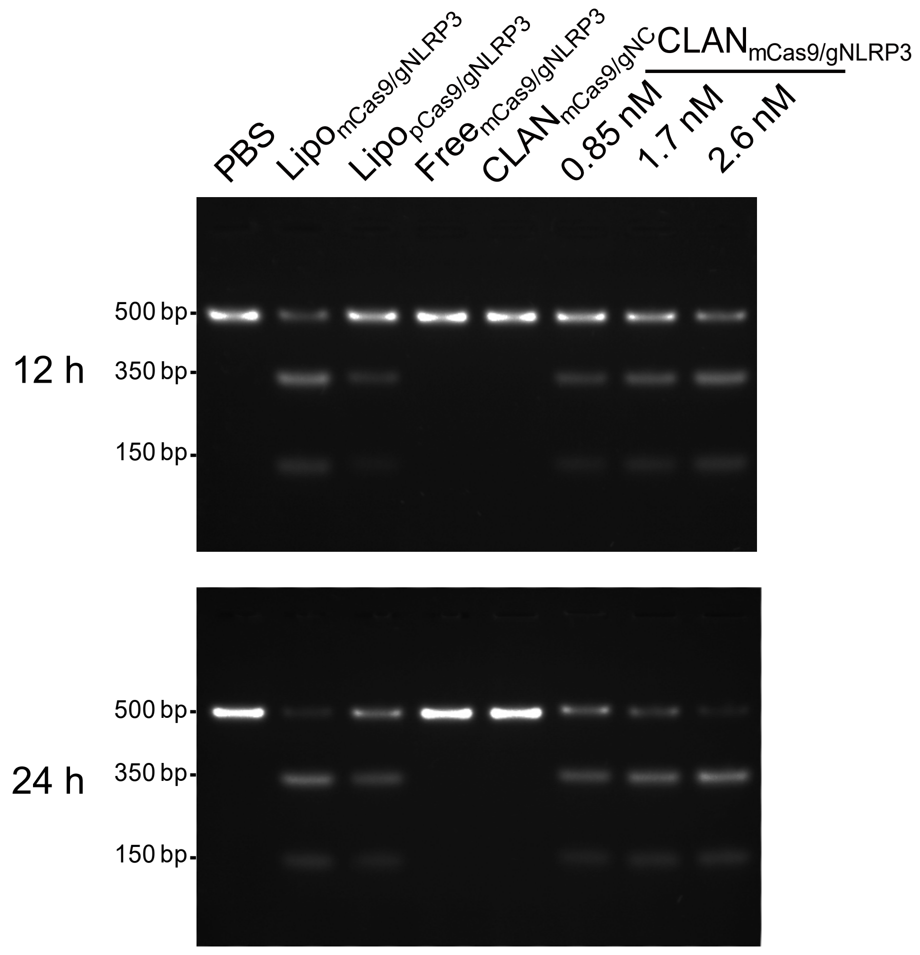

Fig. 2b

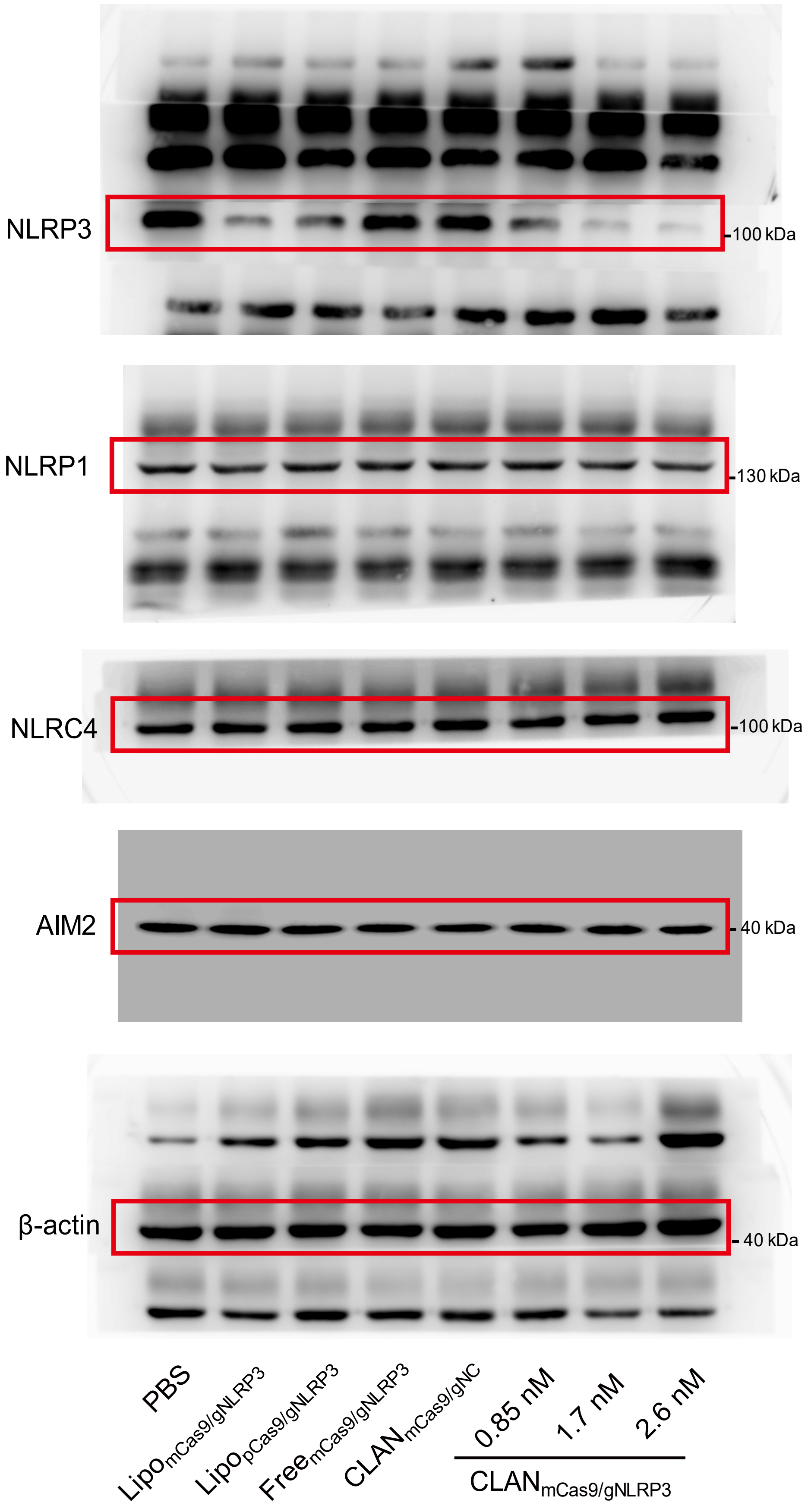

Fig. 2d

Supplementary Fig. 16 Scans of the films used to generate blots data for Fig. 3a and 2f.

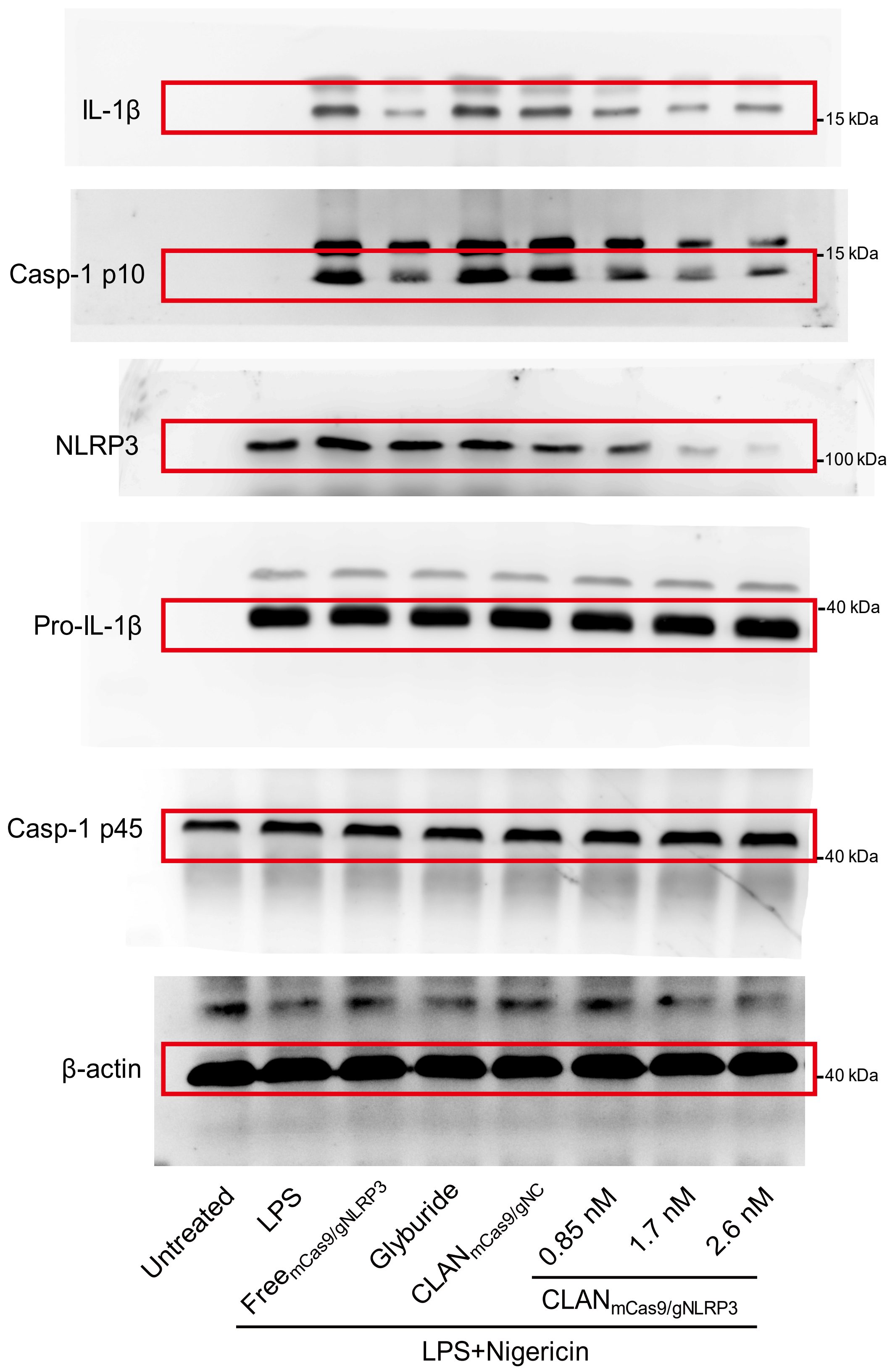

Fig. 3a

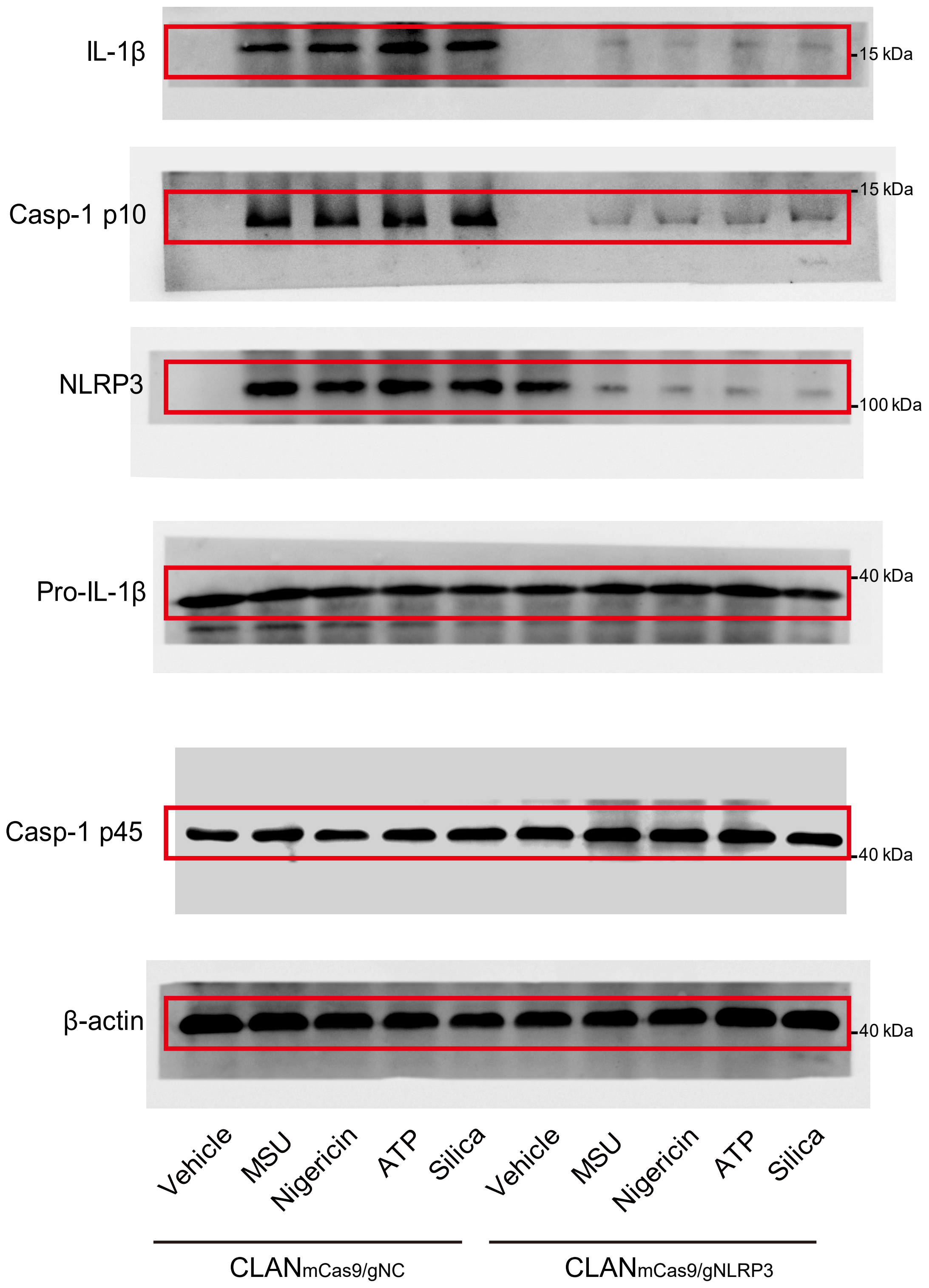

Fig. 3f

Supplementary Fig. 17 Scans of the films used to generate blots data for Fig. 4a, 4b and 4d.

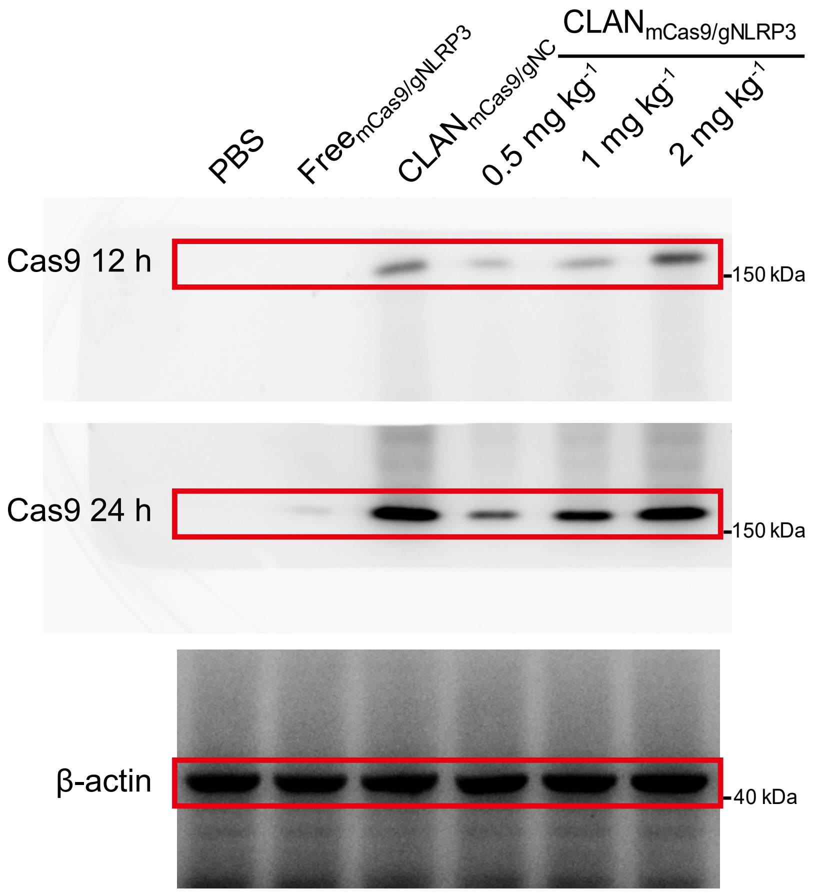

Fig. 4a

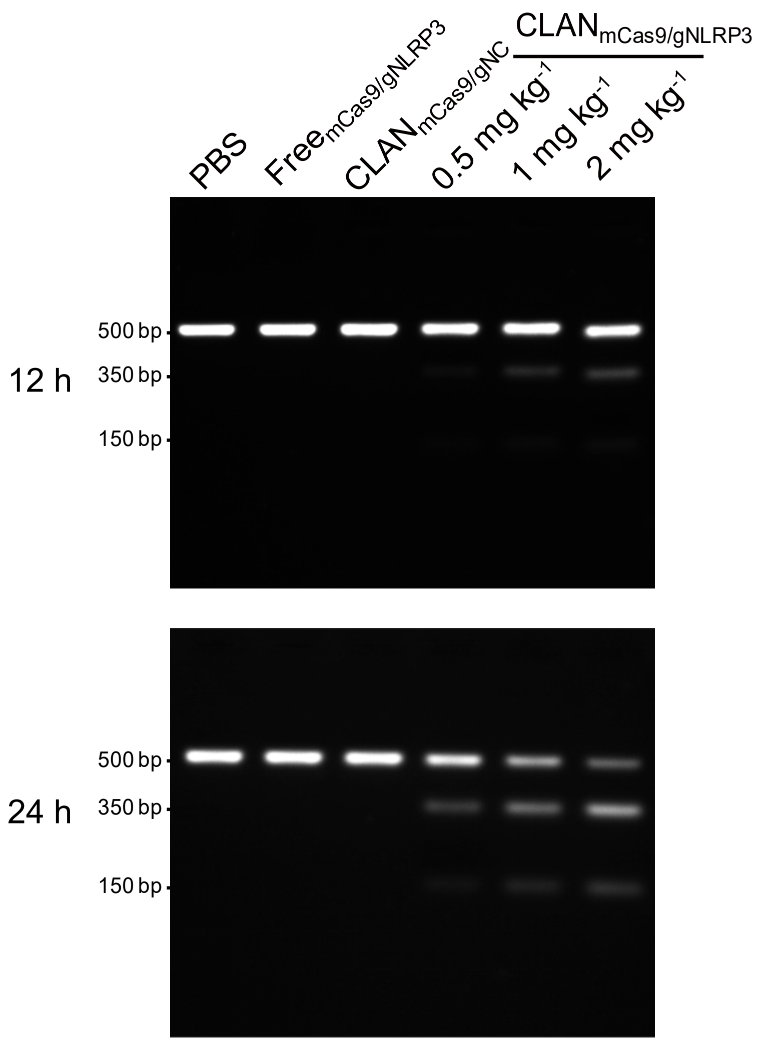

Fig. 4b

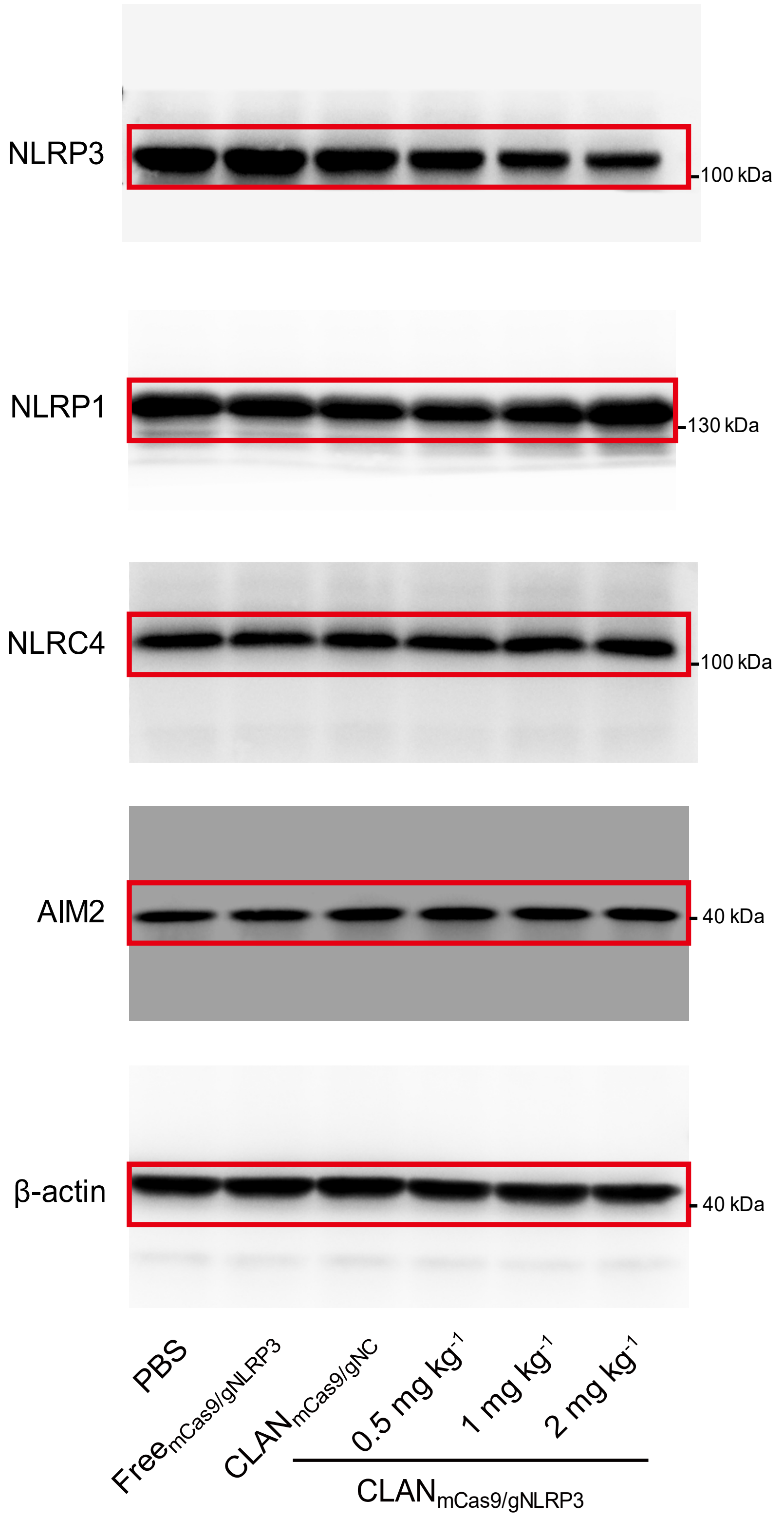

Fig. 4d

Supplementary Fig. 18 Scans of the films used to generate blots data for Fig. 5b, 6d, 7e and Supplementary Fig. 9.

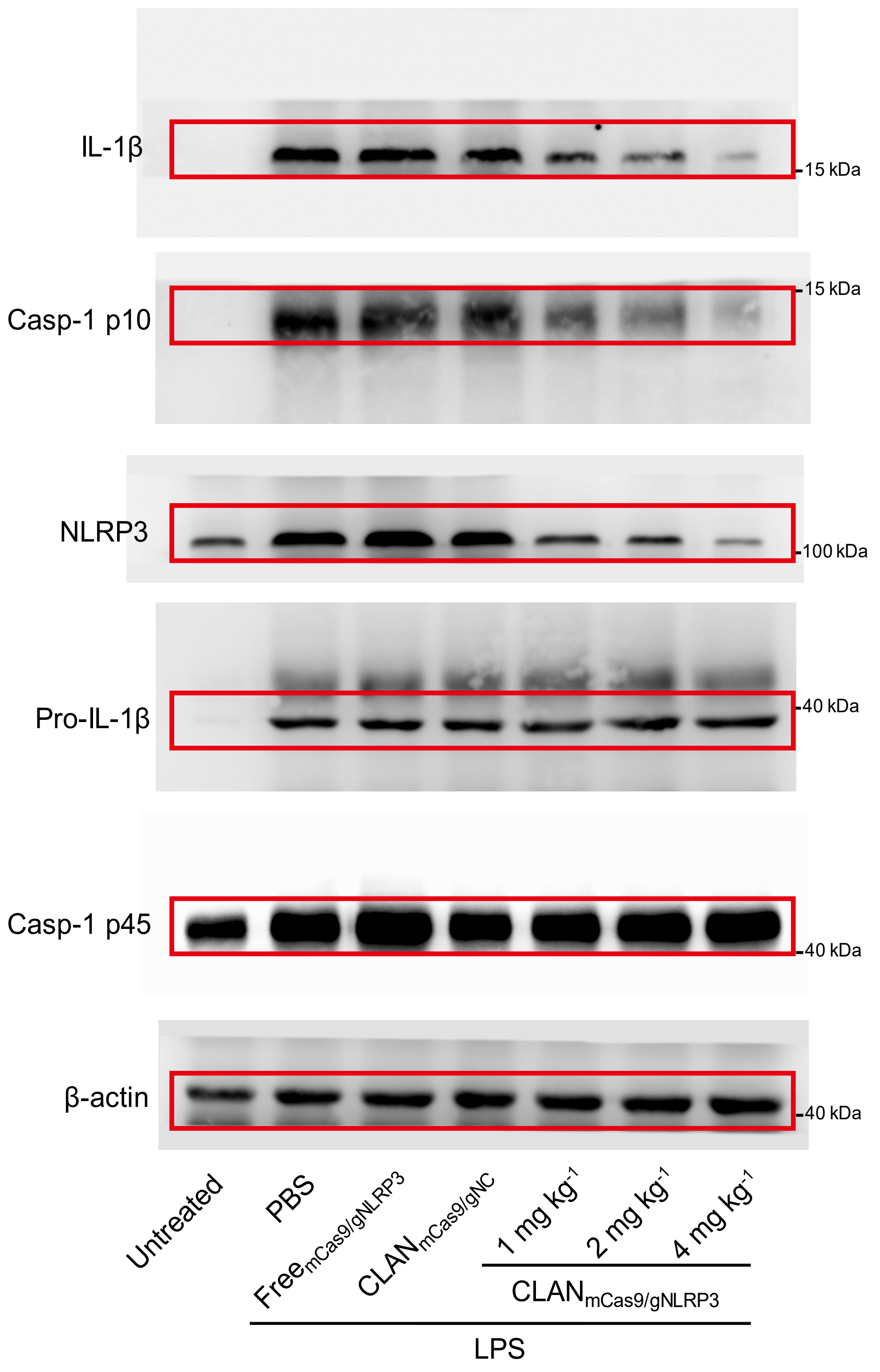

Fig. 5b

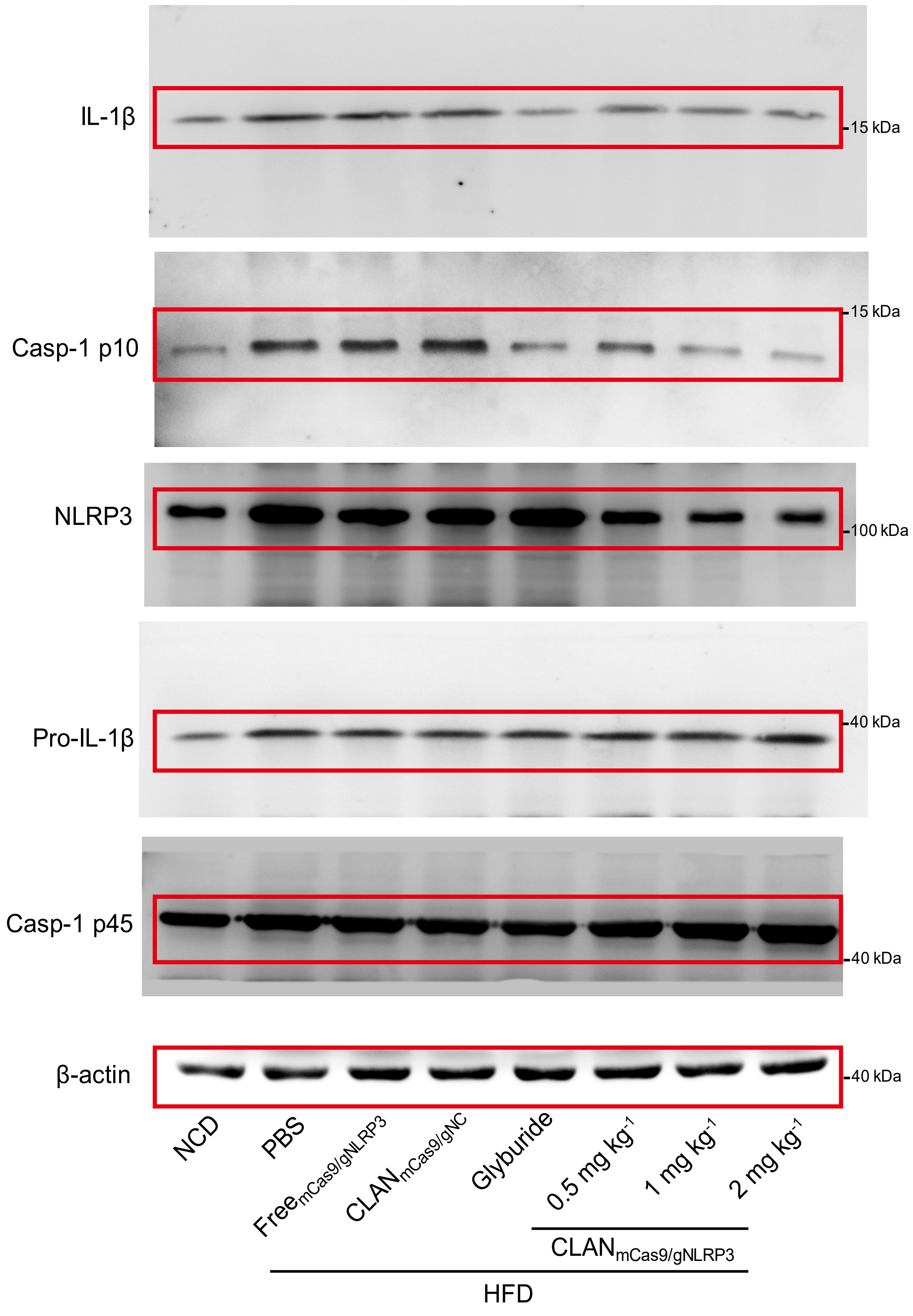

Fig. 7e

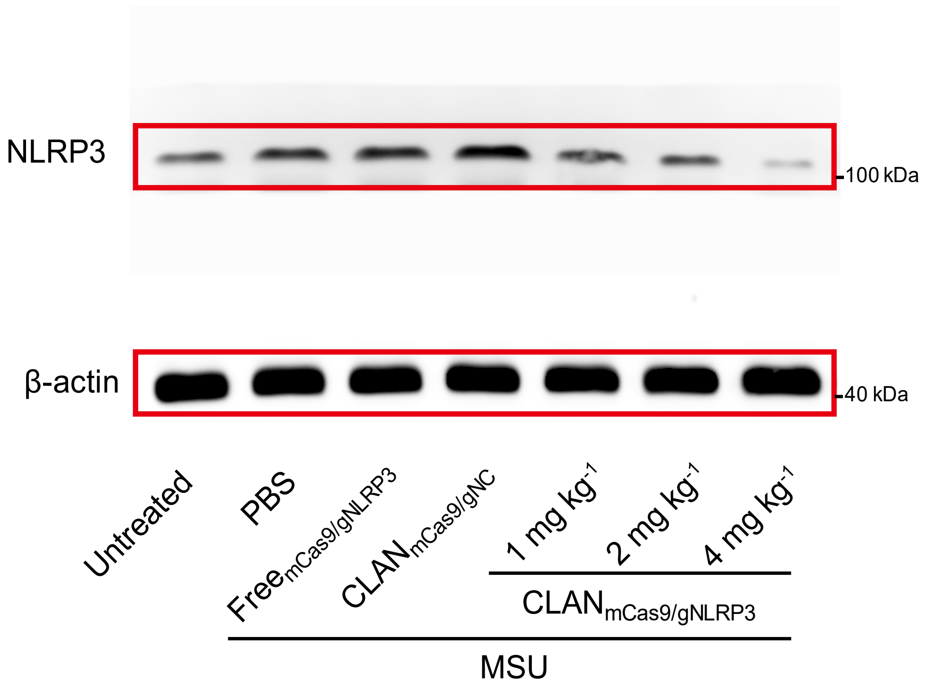

Fig. 6d

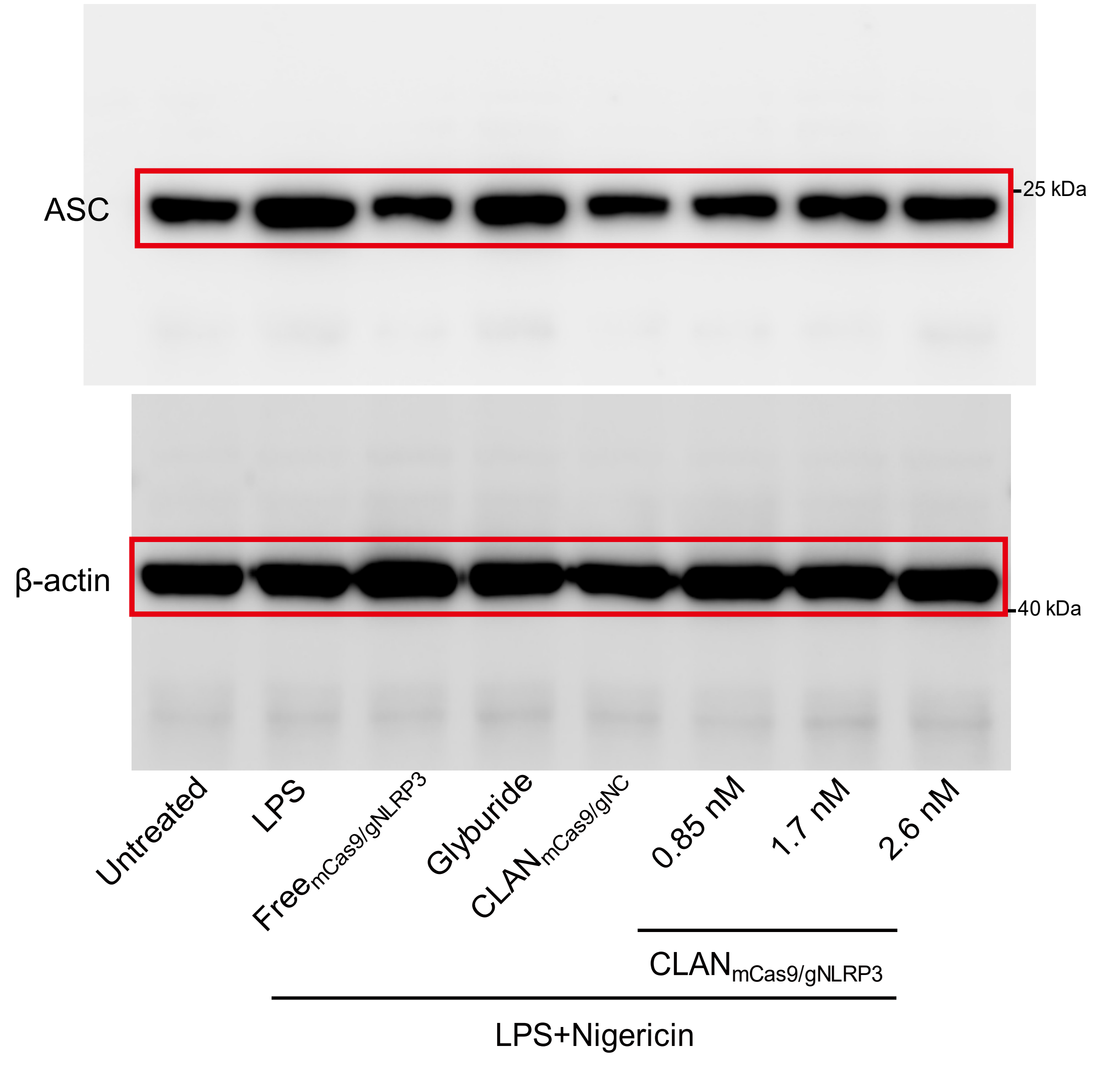

Supplementary Fig. 9
